# Reasons behind permanent tooth extraction at a dental university hospital in Morocco: a survey among patients of the International Faculty of Dental Medicine of Rabat

**DOI:** 10.11604/pamj.2023.46.73.38768

**Published:** 2023-11-02

**Authors:** Rime Chraibi, Hicham Baaddi, Narjiss Akerzoul, Babacar Touré

**Affiliations:** 1 Department of Fixed Prosthodontics, International Faculty of Dental Medicine, College of Health Sciences, International University of Rabat, Rabat, Morocco,; 2 Department of Conservative Dentistry, International Faculty of Dental Medicine, College of Health Sciences, International University of Rabat, Rabat, Morocco,; 3 Department of Oral Surgery-Oral Medicine, International Faculty of Dental Medicine, College of Health Sciences, International University of Rabat, Rabat, Morocco,; 4 Department of Conservative Dentistry, International Faculty of Dental Medicine, College of Health Sciences, International University of Rabat, Rabat, Morocco

**Keywords:** Caries, periodontal disease, avulsion, extraction, oral health, prevention

## Abstract

**Introduction:**

this cross-sectional study aimed to investigate the factors contributing to tooth extractions and possible correlations between tooth loss and various variables.

**Methods:**

the study took place at the dental university hospital affiliated with the International University of Rabat over a period of two months (December 15^th^, 2020, to February 15^th^, 2021). The data collected has been recorded from patients who had tooth extraction procedures. The variables analyzed included age, gender, and the reason for extraction. To analyze the significance of the variables Chi-square test was used, and to investigate the variance in the mean number of teeth extracted per patient ANOVA was used.

**Results:**

in 159 patients with different ages, a total of 586 permanent teeth were extracted. Men had an extraction rate of 54.5% while women formed 45.5%. Overall, the most frequent reason for tooth extraction was caries (46.28%), and periodontal diseases (44.90%). Other reasons for extraction included prosthetics (4.31%) and aesthetics (2.7%). However, orthodontics reasons were not observed. The topmost tooth extraction rate per patient was seen in the 41 to 50-year-old age group with a mean of 4.22 teeth. Analysis of the reasons for extraction by gender did not show a statistically significant difference (p>0.29).

**Conclusion:**

caries and periodontal disease were the main causes of tooth extractions. Improving oral prevention health programs can help patients maintain healthier and functional oral health throughout their lives.

## Introduction

Tooth extraction is the last solution that dental practitioners refer to. There are many factors that can lead to a decrease in the number of teeth, such as dietary habits, oral hygiene, and quality of life [1]. The number of extracted teeth is an indicator of socio-economic and oral hygiene levels. Some reasons behind permanent teeth extractions include dental caries, periodontal disease, orthodontic treatment, impacted teeth, failed dental treatment, and prosthetic indications [2-7]. Determining the reasons of tooth loss can implement effective measures for preventing oral diseases and raising oral health awareness [1]. Previous studies about the reasons behind tooth extraction have shown that dental caries were the leading cause of tooth loss among 20 to 60-year-old patients. Additionally, periodontal diseases were the leading cause of tooth loss among patients in their late 40s and older, as there is an association between age and periodontal disease. In contrast, endodontic treatment complications and orthodontic treatment were the main reasons for tooth extraction among teenagers [8]. Studies have shown that subjects of low income and low education are more prone to be edentulous than those of higher socio-economic status [9-12]. In a study conducted by Ali [9] in 2021 and Jafarian *et al*. [13] in 2013, which studied the relation between gender, level of education, and reasons of tooth loss, it was shown that people who mostly lost their teeth were the poorly educated males. In Morocco, more information is needed on this topic as there is currently a lack of data available. By identifying the main causes and predicting the factors that lead to tooth loss, it may be possible to reduce future extractions. The purpose of this study was to investigate the reasons for permanent teeth extractions and their correlation with factors such as age and gender in Morocco.

## Methods

**Study design:** a cross-sectional study was conducted to determine the reasons behind permanent tooth extraction at a dental university hospital in Morocco.

**Study setting and population:** in Morocco, as in most African countries, the health care needs are high, and the health care structures are limited. Oral diseases are very common and represent a real public health problem in Morocco. On one hand, the Moroccan citizen gives little interest and considers oral care as an expensive and useless prevention. On the other hand, public policy in line with the orientations of the National Strategy for the Development of the Oral Health Sector still lacks efficiency. Morocco has 183 medico-technical equipments in 127 dental centers. More than 300 dentists work in the public sector with a ratio of one dentist per 100,000 people. In Morocco, like many other African countries, there is a high demand for healthcare services while the availability of healthcare facilities is limited. Dental issues are prevalent and make a significant public health concern in Morocco. The general population often neglects oral care, considering it to be expensive and unnecessary for preventative purposes. Furthermore, the public policies that align with the objectives of the National Strategy for the Development of the oral health sector are yet to yield the desired results. Our focus will be on the distribution of dentists in the Rabat-Salé region, where the study was conducted. In Rabat, there are 578 dental surgeons, while Salé has 155 dentists, with only 9 practicing in the public sector and 146 in private practices. Unfortunately, the overall coverage of dentists in the public sector is weak and inadequate, as a result, public sector dentists are often overwhelmed, lacking proper equipment and resources. To address the shortage of dental practitioners, particularly in rural areas, new strategies have been implemented to improve human resources. In Morocco, dentistry training is offered in two cities, Rabat and Casablanca (5 faculties) with plans to establish additional faculties in other locations. The International Faculty of Dental Medicine at the International University of Rabat is located in Rabat-Salé region, it has recently opened a new Dental Clinic. It has 74 chairs, divided into four main areas: emergencies and consultations, oral rehabilitation, orthodontics and pediatric dentistry, and finally oral surgery and periodontology. Patients from Rabat-Salé region frequently visit the university dental clinic for treatment. The study was conducted from December 15, 2020, to February 15, 2021. The data was obtained from 280 undergraduate students and recorded by one examiner.

**Variables:** various variables were examined, including the number of dental trainees involved in the study, as well as the age, gender, address location, tooth type, and reason for extractions of the patients. Additionally, other variables such as age and gender related to tooth extraction was analyzed, and the number of extracted teeth per patient was investigated.

### Data source and measurement

**Data collection tool:** a two-part questionnaire was handed and filled by the dental trainee (4^th^, 5^th^ and 6^th^ year undergraduate students) when taking care of the patients.

**Data collection:** demographic variables (gender, age, level of education, and address) were collected. Also, variables such as the chief complaint (pain, periodontal disease, mobility, esthetics, trauma, other reasons to be specified); the reason for tooth/teeth extraction, including caries (fractured teeth and remaining roots), periodontal disease, endodontic treatment complications (fractures persistent symptoms, resistant periapical healing, and), trauma, pre-prosthetic, orthodontic treatment, and other reasons (esthetic reasons, tooth malposition). Finally, variables such as the type of tooth and the number of extracted teeth per patient were also recorded. Confidentiality and anonymity were maintained throughout the study. The collected data was recorded on a computer

**Sample size:** sample dental trainee size was calculated using Huot's method, a stratified sample of 280 practitioners was chosen as representing the student´s sample size.

**Data analysis:** the data was analyzed using Jamovi 1.8.1. The significance of patient variables such as age and gender related to tooth extraction was analyzed using the Chi-square Test. The variance in the mean number of extracted teeth per patient was investigated using the ANOVA test, and a p-value ≤ 0.05 was considered statistically significant.

**Ethical consideration:** the study design was approved by the Medical and Ethics Committee of the International Faculty of Dental Medicine and the International University of Rabat (CUMD/FIMD 02/021).

## Results

**Sociodemographic analysis:** out of 280 students, 159 participated in this study with a participation rate of 56.78%. The total number of patients in this study was 159, including 45.44% women and 54.56% men. The mean age of patients was 42.7 ± 11.5 years, ranging from 9 to 69 years. 97 patients, representing 61%, live in Salé, the rest came from other surrounding areas (such as Rabat).

**Figure 1:**
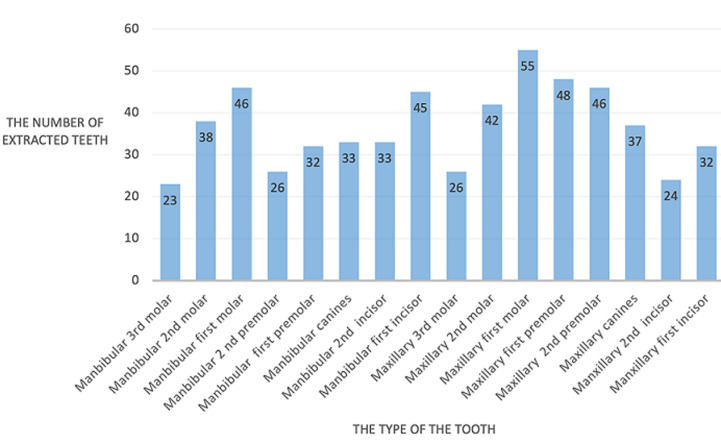
the number of extracted teeth, grouped by their different types

**Figure 2:**
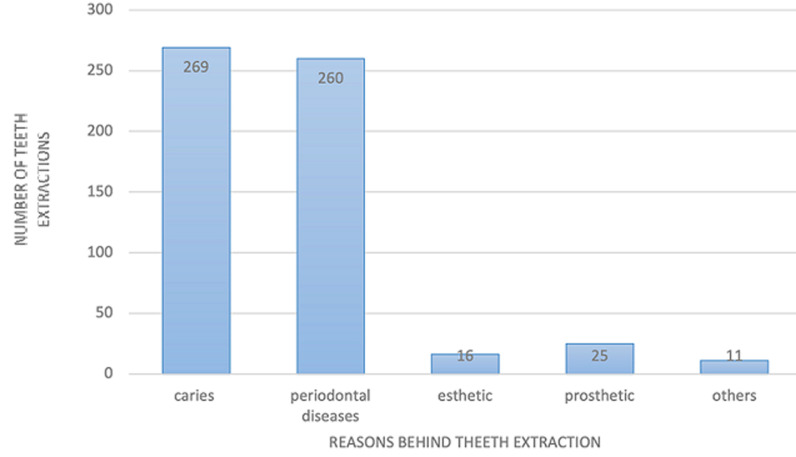
the number of extracted teeth according to the different reasons of extraction

**Main results:** the main chief complaints among the patients were dental pain (62.26%), followed by periodontal disease (21.38%), and esthetics (9.43%). Complaints such as trauma, total crown destruction, prosthetic treatment, and halitosis represented (6.93%) of the sample. The responses of the patients did not show a significant difference based on their place of residence or education level (p= 0.67 and 0.28, respectively). However, periodontal disease was significantly more common among men (79.41%) than among women (20.59%) (p= 0.01). Gender did not significantly affect the prevalence of complaints related to pain or esthetics (p= 0.89 and p= 0.09, respectively). A total of 586 teeth were extracted from 159 patients during the study period (mean extractions per patient = 3.648 ± 3.11 teeth). Males (54.56%) had more missing teeth than females (45.44%). The distribution of patients and extracted teeth by age, range, and gender is presented in Table 1. The molars were the most extracted teeth (39.24%), followed by premolars (25.93%) and incisors (22.9%). Canines were the least commonly extracted teeth (11.94%). The maxillary teeth were extracted more often (52.9%) compared to mandibular teeth (48.1%). According to tooth type, the results showed that the maxillary first molar was the most extracted tooth (9.38%), followed by the maxillary second premolar. The first mandibular molar was in third place, followed by the maxillary premolar (7.84%) and the second maxillary and mandibular molars (7.16% and 6.48%, respectively). The mandibular premolars were extracted less frequently (5.46% for the first premolar and 4.43% for the second premolar). Finally, incisors, canines, and third mandibular molars were the least commonly extracted (Figure 1). Out of the 586 extracted teeth, the reasons for extraction were recorded for 581 of them. Caries and its consequences accounted for 46.28% of extractions, while 44.90% were due to periodontal disease. Other reasons for extraction included removable prosthetics (4.31%), aesthetics (2.7%), and other causes such as endodontic failure. Orthodontic reasons were not noted (Figure 2). The highest rate of tooth extraction per patient was seen in the 41 to 50-year-old age group (mean of 4.22 teeth) and in the 51 to 60-year-old age group (mean of 3.88 teeth) (Table 2). The analysis of extraction reasons by gender did not show a statistically significant difference (p > 0.29).

**Table 1:** distribution of patients and extracted teeth by age range and gender

Age range years	Male patients	Male’s teeth	Female patients	Female's teeth	Total patients	Total teeth
9-20	1	1	4	4	5 (3.14%)	5 (0.86%)
21-30	10	30	8	31	18 (11.32%)	61 (10.49%)
31-40	23	88	27	84	50 (31.44%)	172 (29.60%)
41-50	28	122	17	68	45 (28.30%)	190 (32.70%)
51-60	18	58	16	74	34 (21.38%)	132 (22.71%)
≥ 61	6	18	1	3	7 (4.40%)	21 (3.61%)
**Total**	86 (54.08%)	317 (5456%)	73 (45.91%)	264 (45.43%)	159 (100%)	581

**Table 2:** reasons for tooth extraction by age range

Age range years	Caries	Periodontal disease	Prosthetics	Esthetics	Others*
≤30 (n=66)	24 (36.36%)	37 (56.06%)	-	3 (4.54%)	2 (3.03%)
31-40 (n=172)	89 (51.74%)	72 (41.86%)	2 (1.16%)	2 (1.16%)	7 (4.07%)
41-50 (n=190)	83 (43.68%)	88 (46.31%)	9 (4.73%)	9 (4.73%)	1 (0.59%)
51-60 (n=132)	63 (47.72%)	52 (39.39%)	14 (10.60%)	2 (1.51%)	1 (0.75%)
≥ 61 (n=12)	10 (47.61%)	11(52.38%)	-	-	-
Total (n=581)	269 (46.29%)	260 (44.75%)	25 (1.04%)	16 (0.27%)	11 (0.19%)

* Other reasons: tooth malposition, or the failure of the root canal’s treatment

## Discussion

The present study evaluated the pattern and causes of tooth loss in the university clinic affiliated with the International University of Rabat (UIR) in Morocco. A total 586 teeth were extracted from 159 patients over the study period (mean extractions per patient = 3.648 teeth). Males had more missing teeth than females. Contrary to findings in other countries where females tend to have more tooth loss [12-14]. A similar study conducted in Nigeria and Kuwait found that males had the highest number of tooth extractions due to a lack of interest in adhering to dental maintenance and missing periodic dental visits and because of smoking. In the present study, the highest extraction rate per patient was in the 41 to 50-year-old age group (mean tooth extraction per person of 4.22 teeth) and in the 51 to 60-year-old age group (mean tooth extraction per person of 3.88 teeth). These findings are comparable to other studies [14,15]. Periodontal disease is caused in tooth loss in the 51 to 60-year-old age group. Based on the findings of this study, it can be concluded that caries (46.28%) followed by periodontal disease (44.90%) are the most common reasons for extraction (Figure 2). Nevertheless, tooth extractions were less frequently linked to esthetics 2.7%, prosthetics 4.3%, and endodontic treatment failure especially in older patients. Our findings support the conclusions of other studies in multiple countries including Afghanistan, Greece, Iran, Italy, Kuwait, and Yemen, where caries represented the first reason for tooth extraction followed by periodontal disease [10,12-16]. In some studies, periodontal disease has been found to be a more common cause of tooth loss than tooth decay, and this may be due to factors such as smoking and the presence of chronic diseases. Regarding tooth´s type, molars were the most extracted teeth due to dental caries followed by periodontal diseases, these results were comparable to previous studies from Africa and other parts of the world [10,11,17-19]. Osunde *et al*. [10] in 2017 noted in Nigeria that first and third mandibular molar as well as the maxillary first molar teeth were the most frequently missing teeth. These results were confirmed by Passarelli *et al*. [20] in 2020 and Jafarian *et al*. [13] in 2013, who concluded that the anatomy of the occlusal surface of molars increased their vulnerability to caries. Furthermore, it was found that the first and second molars tended to make eruption at a young age, making them more susceptible to caries and periodontal issues. Extraction of anterior teeth was mainly due to periodontal disease and caries, followed by preprosthetic considerations particularly mandibular anterior teeth in comparison with maxillary anterior teeth. This observation has been previously documented, it is since the anterior mandibular incisors are shielded by the tongue, which makes it less susceptible to tooth decay. However, in elderly population the mandibular anterior area develops gum disease. The least frequently removed teeth are the canines due to its anatomy, smooth crowns, and large roots making them more resistant to both tooth decay and periodontal diseases [12,13,20]. Understanding the patterns of oral diseases in developing countries can help in developing effective preventive measures and treatment strategies for dental health programs.

## Conclusion

The reasons for tooth extraction in this study reflect the prevalence and severity of caries and periodontal disease, which are the most common causes of tooth loss in Morocco. Therefore, improving oral health, particularly through the prevention of dental caries and proper periodontal assessment and treatment, is crucial for patients to maintain a healthy and functional mouth throughout their lives. In addition, further studies involving larger groups are recommended to investigate potential variations in tooth extraction patterns between different regions of Morocco and to examine the possible relationships between tooth loss and risk factors such as chronic diseases and smoking habits.

### What is known about this topic

- *Dental prevention measures have been successful in reducing the incidence of tooth decay and periodontal disease;*
- *Access to a dental care system can also help prevent the causes of tooth loss;*
- *Effective management of chronic health conditions can help prevent the development of periodontal disease and reduce the risk of tooth loss*.

### What this study adds

- *This study provides information about the most common reasons for tooth extraction in Morocco;*
- *There is a need to improve oral prevention efforts and increase access to oral treatment to improve oral health in Morocco*.

## References

[1] Gerritsen AE, Allen PF, Witter DJ, Bronkhorst EM, Creugers NH (2010). Tooth loss and oral health-related quality of life: a systematic review and meta-analysis. Health Qual Life Outcomes.

[2] Noman NA, Aladimi AA, Alkadasi BA, Alraawi MA, Al-Iryani GM, Shaabi FI (2019). Social habits and other risk factors that cause tooth loss: an associative study conducted in Taiz Governorate, Yemen. J Contemp Dent Pract.

[3] Desvarieux M, Schwahn C, Völzke H, Demmer RT, Lüdemann J, Kessler C (2004). Gender differences in the relationship between periodontal disease, tooth loss, and atherosclerosis. Stroke.

[4] Ong G (1996). Periodontal reasons for tooth loss in an Asian population. J Clin Periodontol.

[5] Matthews DC, Smith CG, Hanscom SL (2001). Tooth loss in periodontal patients. J Can Dent Assoc.

[6] Esan TA, Olusile AO, Ojo MA, Udoye CI, Oziegbe EO, Olasoji HO (2010). Tooth loss among Nigerians treated in teaching hospitals: a national pilot study. J Contemp Dent Pract.

[7] Anyanechi C, Chukwuneke F (2012). Survey of the reasons of dental extractions in eastern Nigeria. Ann Med Health Sci Res.

[8] Aida J, Morita M, Akhter R, Aoyama H, Masui M, Ando Y (2009). Relationships between patient characteristics and reasons for tooth extraction in Japan. Community Dent Health.

[9] Ali D (2021). Reasons for Extraction of Permanent Teeth in a University Dental Clinic Setting. Clin Cosmet Investig Dent.

[10] Osunde OD, Efunkoya AA, Omeje KU (2017). REASONS FOR LOSS OF THE PERMANENT TEETH IN PATIENTS IN KANO, NORTH WESTERN NIGERIA. J West Afr Coll Surg.

[11] Alesia K, Khalil HS (2013). Reasons for and patterns relating to the extraction of permanent teeth in a subset of the Saudi population. Clin Cosmet Investig Dent.

[12] Al-Shammari KF, Al-Ansari JM, Al-Melh MA, Al-Khabbaz AK (2006). Reasons for tooth extraction in Kuwait. Med Princ Pract.

[13] Jafarian M, Etebarian A (2013). Reasons for extraction of permanent teeth in general dental practices in Tehran, Iran. Med Princ Pract.

[14] Chrysanthakopoulos NA (2011). Reasons for extraction of permanent teeth in Greece: a five-year follow-up study. Int Dent J.

[15] Da'ameh D (2006). Reasons for permanent tooth extraction in the North of Afghanistan. J Dent.

[16] Morita M, Kimura T, Kanegae M, Ishikawa A, Watanabe T (1994). Reasons for extraction of permanent teeth in Japan. Community Dent Oral Epidemiol.

[17] Ong G, Yeo JF, Bhole S (1996). A survey of reasons for extraction of permanent teeth in Singapore. Community Dent Oral Epidemiol.

[18] Angelillo IF, Nobile CG, Pavia M (1996). Survey of reasons for extraction of permanent teeth in Italy. Community Dent Oral Epidemiol.

[19] Caldas AF (2000). Reasons for tooth extraction in a Brazilian population. Int Dent J.

[20] Passarelli PC, Pagnoni S, Piccirillo GB, Desantis V, Benegiamo M, Liguori A (2020). Reasons for Tooth Extractions and Related Risk Factors in Adult Patients: A Cohort Study. Int J Environ Res Public Health.

